# LCP1 correlates with immune infiltration: a prognostic marker for triple-negative breast cancer

**DOI:** 10.1186/s12865-024-00635-x

**Published:** 2024-07-08

**Authors:** Shuaikang Pan, Mengting Wan, Hongwei Jin, Ran Ning, Jinguo Zhang, Xinghua Han

**Affiliations:** 1 Department of Medical Oncology, The First Affiliated Hospital of USTC, Division of Life Science and Medicine, University of Science and Technology of China, Hefei, China; 2 School of Medical Oncology, Wan Nan Medical College, Wuhu, China; 3 Department of Pathology, The Affiliated Chaohu Hospital of Anhui Medical University, Chaohu, 238000 Anhui China

**Keywords:** LCP1, Triple-negative breast cancer, Prognostic, Biomarker, Immune infiltrate

## Abstract

**Objective:**

Triple-Negative Breast Cancer (TNBC) is known for its aggressiveness and treatment challenges due to the absence of ER, PR, and HER2 receptors. Our work emphasizes the prognostic value of LCP1 (Lymphocyte cytosolic protein 1), which plays a crucial role in cell processes and immune cell activity, to predict outcomes and guide treatments in TNBC.

**Methods:**

We explored LCP1 as a potential biomarker in TNBC and investigated the mRNA and protein expression levels of LCP1. We investigated different databases, including GTEX, TCGA, GEO, cBioPortal and Kaplan-Meier Plotter. Immunohistochemistry on TNBC and benign tumor samples was performed to examine LCP1's relationship with patient clinical characteristics and macrophage markers. We also assessed survival rates, immune cell infiltration, and drug sensitivity related to LCP1 using various bioinformatics tools.

**Results:**

The results indicated that LCP1 expression was higher in TNBC tissues compared to adjacent normal tissues. However, high expression of LCP1 was significantly associated with favorable survival outcomes in patients with TNBC. Enrichment analysis revealed that genes co-expressed with LCP1 were significantly enriched in various immune processes. LCP1 showed a positive correlation with the infiltration of resting dendritic cells, M1 macrophages, and memory CD4 T cells, and a negative correlation with M2 macrophages. Further analysis suggested a link between high levels of LCP1 and increased survival outcomes in cancer patients receiving immunotherapy.

**Conclusion:**

LCP1 may serve as a potential diagnostic and prognostic biomarker for TNBC, which was closely associated with immune cell infiltration, particularly M1 and M2 macrophages. Our findings may provide valuable insights into immunotherapeutic strategies for TNBC patients.

**Supplementary Information:**

The online version contains supplementary material available at 10.1186/s12865-024-00635-x.

## Introduction

Breast cancer (BC), a complex and varied disease, is among the leading types of cancer affecting women worldwide [1]. Recent global cancer statistics from 2021 indicated about 2.3 million new BC cases and roughly 0.69 million BC-related fatalities, surpassing the incidence of lung cancer [2]. BC is commonly classified into three main subtypes based on molecular markers: estrogen receptor (ER), progesterone receptor (PR), and human epidermal growth factor receptor 2 (HER2). These main subtypes are hormone receptor-positive (HR+), HER2-positive, and triple-negative breast cancer (TNBC) [3]. Notably, TNBC accounts for 15%-20% of all BC cases and is characterized by its aggressive behavior, poorer prognosis, and limited therapeutic options [4]. For TNBC patients, conventional endocrine or HER2-targeted therapies are ineffective due to the absence of specific receptor markers. As a result, the primary non-surgical treatment for TNBC is standard chemotherapy [5]. TNBC shows a relatively better response to typical chemotherapy regimens, such as taxanes or anthracyclines. However, complete remission is achieved in fewer than 30% of TNBC patients, and recurrence and mortality rates are higher compared to other BC subtypes [6].

In the realm of tumor treatment, immunotherapies, particularly immune checkpoint inhibitors, have shown promising developments in recent years [7, 8]. BC was traditionally viewed as a poor candidate for its low tumor mutational burden and limited production of tumor neoantigens [9]. Yet, emerging research underscores a significant interplay between BC and the immune system [10]. According to extensive clinical studies, immune checkpoint inhibitors (ICIs) such as pembrolizumab and atezolizumab have demonstrated efficacy in treating early-stage or metastatic TNBC [11, 12]. Nevertheless, the response to ICIs varies significantly among BC patients, both in terms of effectiveness and adverse effects. Additionally, several novel immune-based therapies for BC are still in their nascent stages of clinical development [13]. Hence, there is a compelling need to identify, validate, and harness reliable biomarkers for predicting responses to immunotherapies.

Lymphocyte cytosolic protein 1 (LCP1) is an actin-binding protein initially identified in human fibroblast neoplasm as a member of the fibrin family of phosphoproteins. LCP1 is integral to cellular adhesion, actin binding, and the facilitation of actin assembly, which has been shown to contribute significantly to the invasiveness of tumors and transformed cell lines, highlighting its potential role in oncogenesis [14, 15]. The functions of LCP1 can extend to the immune system, affecting the behavior of various immune cells, such as macrophages, neutrophils, B-cells, and T-cells [16–19]. The association of LCP1 with immune system has sparked interest in its potential as a biomarker for cancer diagnosis and prognosis. In fact, LCP1 has been identified as a prognostic marker in oral, colon, kidney and gastric cancers [20–24]. In BC, the expression of LCP1 can shield cells from TNF-induced apoptosis and facilitate cellular invasion. Consequently, LCP1 may play a dual role in tumor progression and thwarting cell death signals, potentially serving as an effective prognostic marker [25]. This associations may underscore the importance of LCP1 as a potential prognostic biomarker in TNBC and its role in immune infiltration.

In this research, we embarked on a comprehensive investigation of LCP1diverse functions in TNBC. First, we analyzed LCP1 expression levels in both human normal and TNBC tissues, utilizing data from public databases. We further validated the levels of LCP1 protein expression in both TNBC and normal tissue samples through immunohistochemistry. Additionally, we assessed the expression levels of macrophage markers CD80 and CD206, which are indicative of M1 and M2 phenotypes, respectively. We also explored the correlations between LCP1 expression and survival rates, as well as clinicopathological characteristics, and evaluated LCP1 as a potential prognostic biomarker using the Cox regression model. Moreover, the study delved into the association between LCP1 and various immune cell types, especially M1 and M2 macrophages, and its potential influence on immune cell infiltration. Overall, our study endeavors to deliver a thorough understanding of LCP1complex roles in TNBC, highlighting its potential as a predictive biomarker in the clinical setting.

## Materials and methods

### LCP1 expression in human normal tissues and TNBC

To analyze LCP1 expression in 31 different healthy human tissues, We extracted a total of 7,858 normal tissue samples via a Perl script from the GTEx database (https://www.gtexportal.org/home), comprising 4,904 samples from males and 2,954 samples from females. For microarray data, GSE38959 (17 normal and 30 TNBC samples) and GSE65194(11 normal and 55 TNBC samples)were retrieved from the GEO (http://www.ncbi.nlm.nih.gov/geo) (Gene Expression Omnibus) database in the form of MINiML files [26, 27]. We then performed statistical comparisons between two groups using the Wilcox test. The UALCAN tool (http://ualcan.path.uab.edu) was used to analyze the mRNA and proteomic expression of LCP1 across different breast cancer subtypes [28]. The expression level of LCP1 was normalized to transcripts per million reads, and differences with a *P* value of less than 0.05, determined by Student’s t-test, were considered significant. Additionally, we utilized the TISIDB database [29](http://cis.hku.hk/TISIDB/index.php) to explore LCP1 expression across various BC subtypes (*n*=1081). Statistically significant differences were defined as *P*-value < 0.05.

### Genomic alterations of LCP1 in BC

We conducted an in-depth analysis of LCP1 genomic profiles in BC utilizing the cBioPortal database (http://www.cbioportal.org/) [30]. Our study utilized the dataset of breast invasive carcinoma (TCGA, Firehose Legacy) for a comprehensive analysis, extracting 960 samples. The genetic alterations of LCP1 within the TCGA-BRCA dataset were meticulously summarized using the "OncoPrint" module. Furthermore, to explore the intricate relationships involving LCP1 mutations, we employed the "Cancer Type Summary" and "Comparison/Survival" modules for co-occurrence analysis.

### Immunohistochemical staining (IHC) and analysis

The human subject research obtained approval from the Ethics Committee of Chaohu Hospital, Anhui Medical University (KYXM-202212-011). This study involved the retrieval of 11 cases of benign breast tissue and 29 cases of TNBC tissue from patients who had been treated at Chaohu Hospital, Anhui Medical University, from January 2017 to October 2022. These patients had not received radiotherapy or chemotherapy before surgery. Breast tissue samples, which were fixed in 10% formalin and embedded in paraffin, were pathologically diagnosed by clinical pathologists at Chaohu Hospital. The tissue sections were deparaffinized in xylene and then rehydrated in a descending alcohol series. Endogenous peroxidase activity was quenched with 3% hydrogen peroxide for 15 minutes. The sections were blocked with 10% normal goat serum at room temperature for 40 minutes and then incubated overnight at 4°C with anti-LCP1 antibody (13025-1-AP, Proteintech), anti-CD206 antibody(ab64693 ,Abcam) and anti-CD80 antibody(ab134120, Abcam). On the following day, the tissue sections were incubated at room temperature for 1 hour with biotinylated anti-rabbit secondary antibody (Maixin Bio). After washing, signal detection was performed using a DAB kit (Maixin Bio). Finally, the section was counterstained with hematoxylin and photographed under a light microscope. A normal breast tissue without a primary antibody was used as a negative control. The scoring of LCP1, CD80 and CD206 immunoreactive staining was conducted by two independent pathologists who were blinded to the patients' clinical data. Based on the intensity of cell staining, the scoring is divided into four levels: no positive staining (negative) scores 0 points, light yellow (weak positive) scores 1 point, brown-yellow (positive) scores 2 points, and dark brown (strong positive) scores 3 points. In this study, we define cases with an Immunohistochemistry (IHC) score of 0 as negative expression, and cases with an IHC score greater than 0 as positive expression.

### Correlation of LCP1 expression with patient survival outcomes and clinical pathological parameters

Using the Kaplan-Meier Plotter database (www.kmplot.com) [31], we assessed the prognostic value of LCP1 for the survival outcome of TNBC patients. Based on the median mRNA expression level of LCP1, all patients were divided into two groups: high expression and low expression. Survival analysis plots, hazard ratios, 95% confidence intervals, and log-rank *P* values were then examined. We calculated the LCP1 expression for overall survival (OS) of breast cancer (*n*=1879), as well as its correlation with the OS (*n*=404), recurrence-free survival (RFS) (*n*=846), post-progression survival(PPS) (*n*=66),and distant metastasis-free survival (DMFS) (*n*=671) of the Basal subtype using automatically selected optimal cutoff values. Clinical-pathological parameters, including age, stage, tumor size, and lymph node involvement, were extracted from the TCGA-TNBC cohort (*n*=123). We analyzed the correlation between LCP1 expression and clinical-pathological factors.

### Differential gene expression (DEGs) screening and enrichment analysis

We employed the "limma" R package to discern differentially expressed genes (DEGs) within the TCGA-TNBC cohort, comparing the high expression group to the low expression group of LCP1 [32]. DEGs meeting the criteria of an absolute log2-fold change (FC) > 1 and an adjusted *p*-value < 0.05 were selected for subsequent analysis. Subsequently, we conducted Gene Ontology (GO) and Kyoto Encyclopedia of Genes and Genomes (KEGG) pathway analyses on the DEGs using the R software package "ClusterProfilter" [33], an adjusted *P* < 0.05 was considered statistically significant. Multiple gene set enrichment analysis (GSEA) plots were generated utilizing the MSigDB categories (h.all.v7.5.1.symbols.gmt and c2.cp.kegg.v7.4.symbols.gmt) via the R packages "enrichplot" and "clusterProfiler." In this analysis, we only present the top 8 terms meeting the criteria of a *P*-value < 0.05 and a q-value < 0.25.

### Correlation between LCP1 expression and infiltration of immune cells

We investigated the connection between LCP1 expression and the levels of various infiltrating immune cells using CIBERSORT and ESTIMATE algorithms [34, 35]. Then, we used the TIMER 2.0 database(http://timer.cistrome.org/) to analyze the correlation between LCP1 expression and the presence of M1 and M2 macrophages in TNBC (*n*=191) [36]. We utilized the "corrplot" package to further explore Spearman correlations between LCP1 and immune checkpoint genes. Additionally, we analyzed the associations between LCP1 and chemokine receptors, as well as chemokines, using TISIDB (*n*=1100 ) (http://cis.hku.bhk/TISIDB/).

### Drug sensitivity analysis and immunotherapy response

We employed the R package "pRRophetic" to anticipate the responsiveness of chemotherapy in TNBC by leveraging the extensive pharmacogenomics dataset from the Genomics of Drug Sensitivity in Cancer (GDSC) [37]. In order to assess the connection between LCP1 and drug sensitivity, we utilized regression techniques to calculate the IC50 values for various chemotherapeutic agents. The Immunophenoscore (IPS) proves to be a more effective indicator of the effectiveness of both anti-CTLA-4 and anti-PD-1 therapies [38]. we harnessed immunotherapy cohorts to project the impact of LCP1 on patient survival via the Kaplan-Meier Plotter tool [39].

### Statistical analysis

Data processing, the creation of plots, and statistical analysis were carried out using R software (version 4.2.3, Vienna, Austria). To assess the correlation between LCP1 expression and clinicopathological characteristics, we employed a Fisher's exact test. When comparing two independent groups of data, Student's t-tests were utilized. The *p*-value for the Logrank Test in the survival analysis was calculated using an online tool. Details of other statistical methods are provided in the previous sections.

## Results

### LCP1 expression in normal and TNBC tissues: insights from comprehensive analyses

**Fig. 1:**
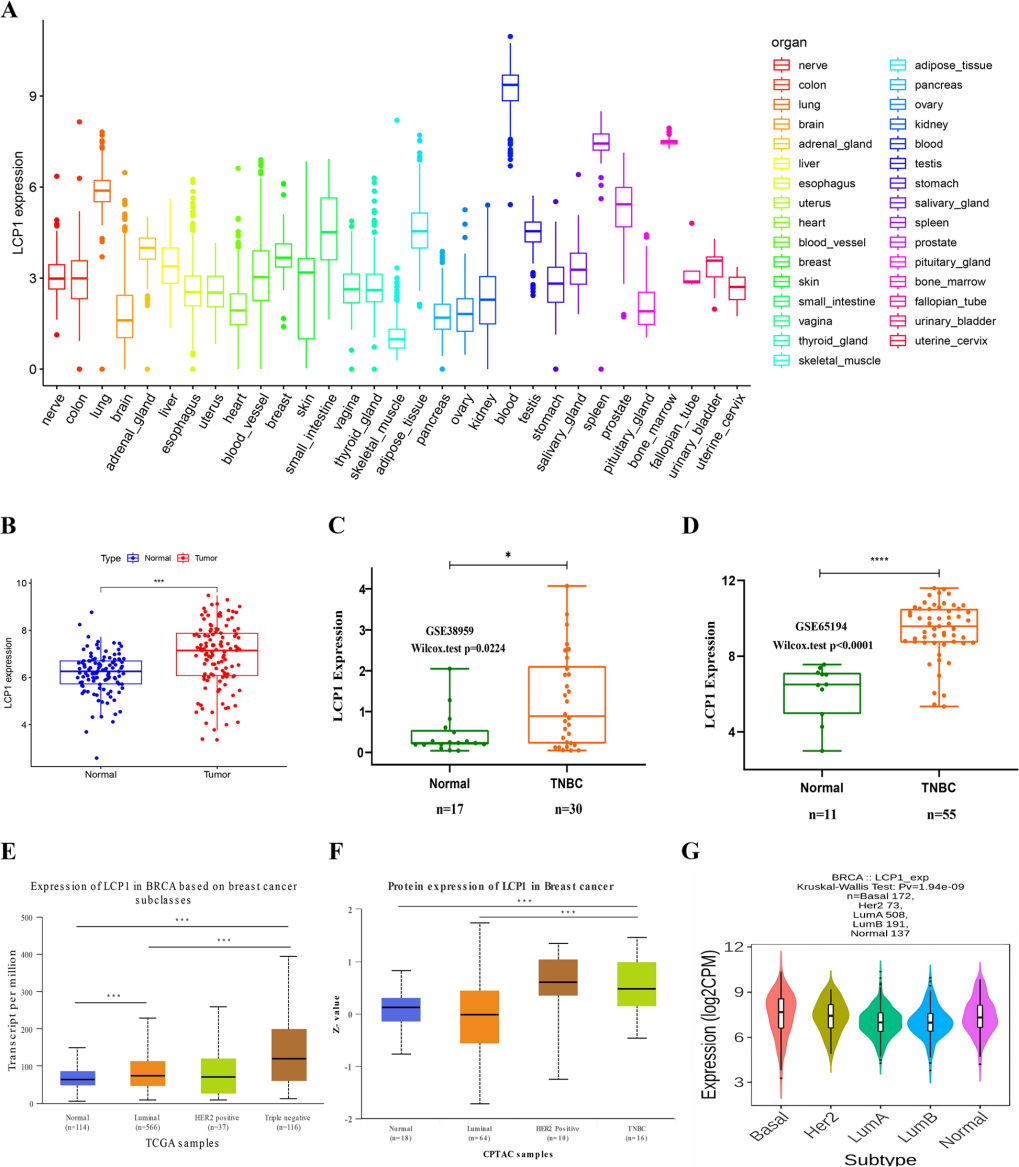
The mRNA level of LCP1 in normal tissues and TNBC. **A** The LCP1mRNA level in human normal tissues using Genotype Tissue-Expression (GTEX) data. **B** Comparison of the LCP1 mRNA level in TCGA-TNBC and TCGA normal breast tissues. The red dots represent the cancer samples, and the blue dots represents the normal samples. **C**, **D** Comparison of the LCP1 mRNA level in TNBC tissues and normal tissues validated by GSE38959 and GSE65194 datasets. E The mRNA level Expression of LCP1 in BRCA based on breast cancer subclasses(Normal-vs-Luminal *P*= 3.58529999999746E-05, Normal-vs-TNBC* P*= 7.85030040972856E-10; Luminal-vs-TNBC *P*= 2.06219999999702E-05). **F** LCP1 proteomic expression profile based on Major subclass using data from Clinical Proteomic Tumor Analysis Consortium (CPTAC) and the International Cancer Proteogenome Consortium (ICPC) datasets(Normal-vs-TNBC *P*=1.705679E-02;Luminal-vs-TNBC *P*= 3.499659E-03). G The mRNA level of LCP1 in different breast cancer subtypes in TCGA database.*, *P* < 0.05; **, *P* < 0.01; and ***, *P* < 0.001

In order to explore the expression pattern of LCP1 under normal physiological conditions in humans, LCP1 expression data were extracted from the GTEX dataset of normal human tissues. LCP1 exhibited higher expression levels in the blood, spleen, and lung tissues, while lower expression levels were found in skeletal muscle (Fig. 1A). Further analysis revealed significant gender differences in LCP1 expression in the brain, thyroid, and adipose tissues, with no significant difference between male and female expression in breast tissue (Supplementary Fig. 1). At the genome level, the LCP1 gene was altered in 39 BC patients, which accounted for only 4% of the 960 samples. The most common alterations of the LCP1 gene in BC were low mRNA expression, high mRNA expression, and deep deletion. Intriguingly, the co-occurrence of mutations such as HTR2A, CBY2, CPB2, and SIAH3 was observed in the LCP1 alteration group (Supplementary Fig. 2). Subsequently, the study focused on exploring the expression of LCP1 in TNBC. In the TCGA-TNBC RNA sequencing data, LCP1 expression levels were higher in tumor tissues compared to normal breast tissues (*P*< 0.001) (Fig. 1B). To validate the expression results from TCGA, GSE38959 and GSE65194 were further analyzed, showing consistent results with the TCGA database with significantly higher expression of LCP1 in TNBC tissues compared to normal tissues (Fig. 1C, D). Additionally, among different breast cancer subtypes, the highest expression of LCP1 was observed in the basal subtype(Fig. 1E,G). Compared to normal and Luminal subtypes, the protein levels of LCP1 were also elevated in TNBC (Normal-vs-TNBC *P*=1.705679E-02;Luminal-vs-TNBC *P*= 3.499659E-03) (Fig. 1F).

### LCP1 expression in TNBC: prognostic insights and clinical correlations

**Fig. 2:**
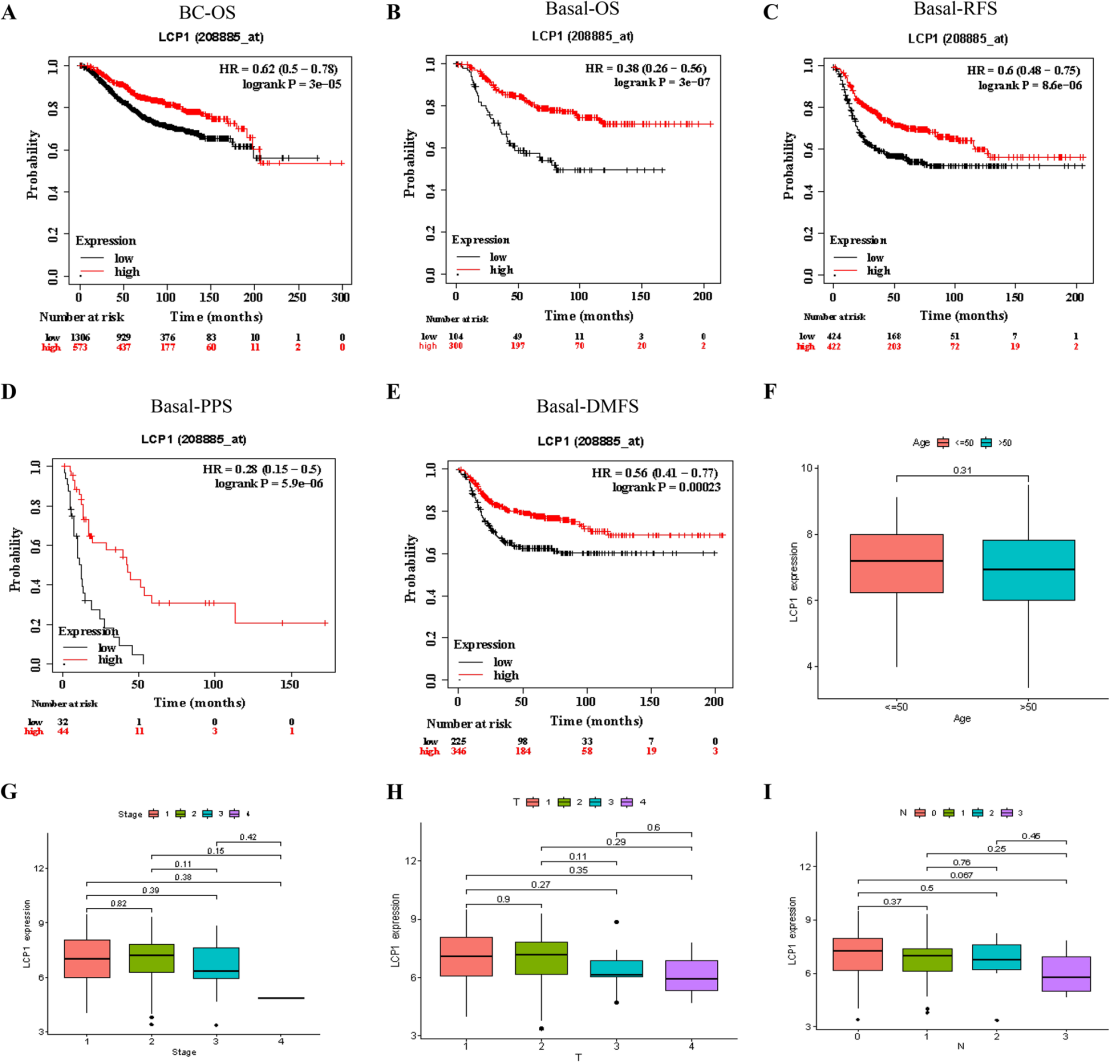
Clinical Implications of LCP1 in TNBC. **A**-**B** Kaplan–Meier survival curves illustrating the impact of LCP1 expression on overall survival in both broad BC and basal-like BC patient groups (Probe ID: 208885_at). **C** Kaplan–Meier analysis assessing relapse-free survival in basal-like BC patients in relation to LCP1 expression. **D** Kaplan–Meier curve depicting post-progression survival in basal-like BC patients, correlated with LCP1 expression. **E** Kaplan–Meier survival analysis for distant metastasis-free survival in basal-like BC patients, linked to LCP1 expression. Patients with expression levels above the median are shown in red, and those below the median in black. HR stands for hazard ratio. **F**-**H** Evaluation of the relationship between LCP1 expression and various clinical parameters (age, stage, tumor size, and node status) in TNBC patients

We next validated the association between LCP1 gene expression levels and the prognosis of patients with TNBC. Kaplan-Meier survival curve analysis was performed for the overall survival (BC-OS HR=0.62 *P*<0.001), overall survival for basal-like subtypes (Basal-OS HR=0.38 *P<0.001*), and relapse-free survival for basal-like subtypes (Basal-RFS HR=0.6 *P*<0.001) across the entire breast cancer cohort. In all these groups, patients with low LCP1 expression levels demonstrated significantly shorter survival times and higher recurrence rates (Fig. 2A-C). Similarly, post-progression survival (Basal-PPS HR=0.28 *P<0.001*) and distant metastasis-free survival (Basal-DMFS HR=0.56 *P<0.001*) both showed significantly reduced survival times in the low-expression group (Fig. 2D-E), emphasizing the potential of LCP1 as a positive prognostic biomarker for patients with basal-like breast cancer. The analysis of the relationship between LCP1 expression levels and patient age, cancer staging, tumor size (T grade), and lymph node involvement (N grade) (Fig. 2F-I) revealed that the association of LCP1 expression levels with patient age, cancer stage, tumor size, and lymph node status was not very pronounced. However, a trend was observed where later stages of tumor had lower LCP1 expression, suggesting that further validation with additional samples might be necessary due to insufficient sample size. Subsequently, a chi-square test analysis of 29 TNBC patients and 11 patients with benign tumors was conducted to examine the relationship between LCP1 immunohistochemical scores and age (*P*=0.66), stage (*P*=0.639), histological type (*P*=0.083), lymph node metastasis (*P*=0.715), and size (*P*=0.450),. The results were consistent with analyses from the TCGA database, showing no significant differences (Table 1).

**Table 1:** Clinicopathological features of patients correlated with LCP1 expression detected by immunohistochemistry

Clinicopathological features	n	LCP1 Expression		*P*-value
		Negative (%)	Positive (%)	
Age at diagnosis				0.66
≤50	14	4(28.6)	10(71.4)	
>50	15	10(66.7)	5(33.3)	
Histological type				0.083
TNBC	29	14(48.3)	15(51.7)	
Benign breast lesions	11	2(18.2)	9(81.8)	
Tumor size				0.450^a^
≤2cm	10	6 (60.0)	4 (40.0)	
>2cm	19	8 (42.1)	11 (57.9)	
LN metastasis				0.715
YES	13	7 (53.8)	6 (46.2)	
NO	16	7 (43.7)	9 (56.3)	
Clinical stage				0.639^b^
I+II	23	11 (47.8)	12 (52.2)	
III	6	3 (50.0)	3 (50.0)	

*TNBC* Triple-negative breast cancer, *NAT* Normal adjacent tissue, *n* Number of cases, *LN* Lymph node

For comparison of LCP1 protein expression associated with age, tumor size, histological type, lymph node metastasis and clinical stage. Pearson's chi-squared test, Fisher's exact test and Continuous modified chi-square test were applied

^a^Tumor size comparison (≤2 cm vs. >2 cm, TNBC cohort)

^b^Clinical stage comparison (I+II vs. III,TNBC cohort)

### Functional enrichment analysis of LCP1 in TNBC

**Fig. 3:**
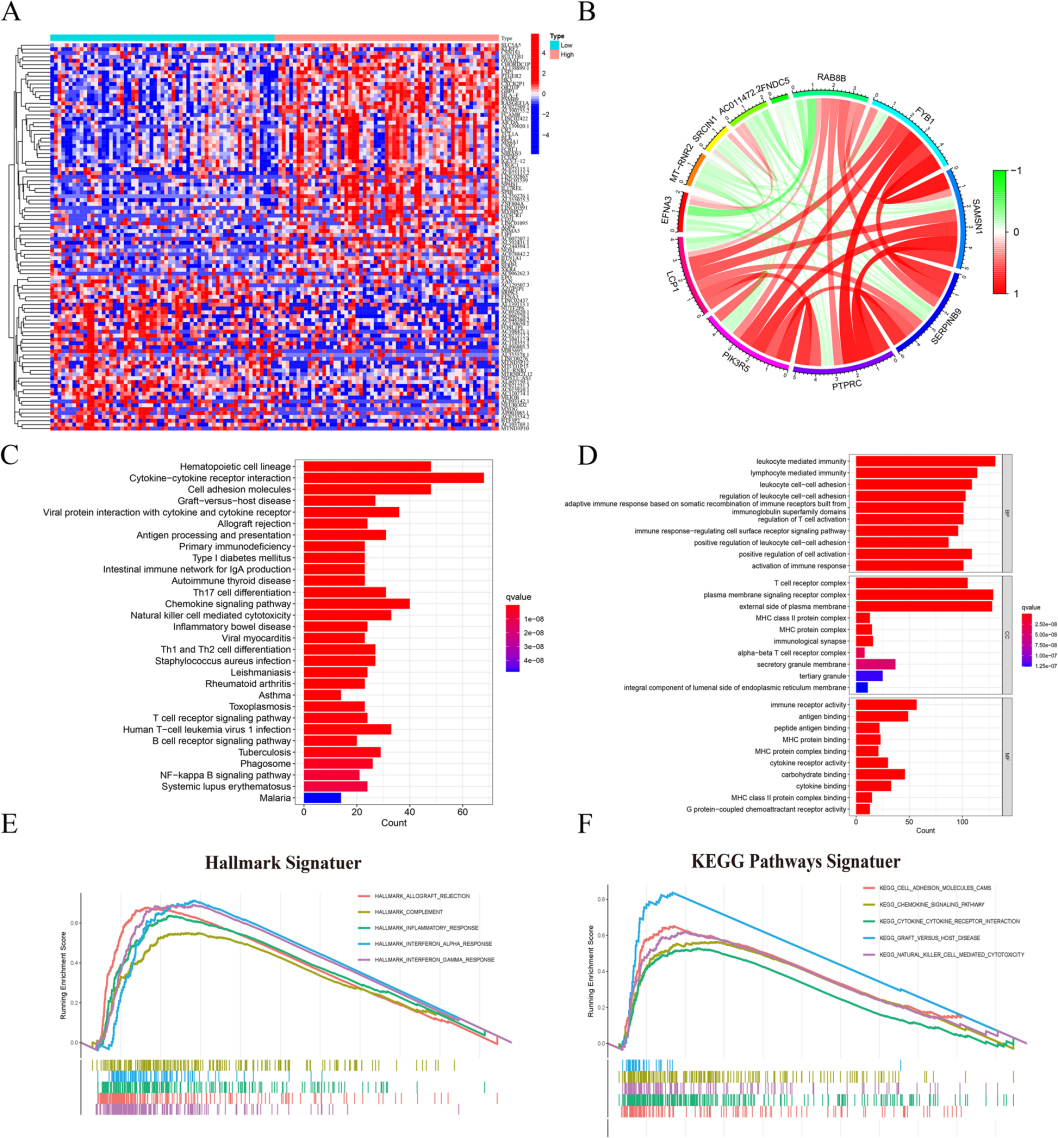
In-Depth Analysis of LCP1's Role in TNBC. **A** A heatmap depiction illustrating the variance in gene expression associated with different levels of LCP1 expression. (Red indicates that gene expression is relatively upregulated, while blue indicates that gene expression is relatively downregulated). **B** A circos diagram displaying genes that are coexpressed with LCP1, emphasizing their interconnectedness in TNBC. **C** An analysis of the KEGG pathways, showing the influence of varying LCP1 expression on differentially expressed genes (DEGs). **D** An exploration of GO terms related to DEGs, broken down by LCP1 expression levels, in the areas of Biological Process (BP), Molecular Function (MF), and Cellular Component (CC) (All terms are color-coded according to the adjusted *P* value, and the length of each bar represents the number of genes). **E** Gene Set Enrichment Analysis (GSEA) focusing on biological processes linked to LCP1 within the framework of the HALLMARK gene set. **F** GSEA illustrating LCP1-related biological processes as per the KEGG gene set, with pathways distinguished by varying color schemes

To better understand the key functions of LCP1 in TNBC, we undertook functional enrichment analyses. Initially, we identified genes exhibiting differential expression in association with LCP1 in TNBC cases. The analysis revealed significant upregulation of genes such as SLC515, LSP1, GBP1, and HLA-F in the high LCP1 expression group, while genes like LINC02437, FOSL1P1, and MYOG were notably downregulated in the same group (Fig. 3A, Supplemental Table 1). Co-expression analysis further revealed a positive correlation of genes such as PIK3R5, PTPRC, SERPINB9, SAMSN1, FYB1, and RAB8B with LCP1 expression, whereas EFNA3, MT-RNR2, SRCIN1, AC011472.2, and FNDC5 showed a negative correlation (Fig. 3B). Our study also involved in-depth analysis of KEGG pathways and Gene Ontology (GO), thoroughly examining the function of LCP1 s. KEGG pathway analysis revealed the important role of LCP1 across numerous biological pathways, particularly highlighting its key role in immune signaling, as evidenced by its significant expression in the cytokine-cytokine receptor interaction pathway. The study also identified active involvement of LCP1 in the hematopoietic cell lineage pathway and the cell adhesion molecules (CAMs) pathway (Fig. 3C). GO analysis underscored the crucial function of LCP1 within the immune system. Data indicated that LCP1 plays a pivotal role in key biological processes such as leukocyte-mediated immunity and lymphocyte-mediated immunity. Moreover, this research disclosed the association of LCP1 with specific cellular components, such as the plasma membrane signaling receptor complex, crucial for T-cell activation. The molecular function of LCP1, particularly its immune receptor activity and cytokine binding capability, further emphasizes its role in the functionality of the immune system (Fig. 3D). In single-gene GSEA analysis, LCP1 expression was predominantly enriched in several key pathways within the HALLMARK signaling pathway, covering important areas such as allograft rejection, complement activation, inflammatory response, interferon-alpha response, and interferon-gamma response. Enrichment maps revealed significant peaks in these pathways, clearly indicating a strong correlation between LCP1 expression and these immune-related pathways. The leading edge subsets within these peaks further revealed the potential role of LCP1 in cellular responses to immune and inflammatory stimuli (Fig. 3E). Additionally, LCP1 also showed significant enrichment in numerous key KEGG pathways, including cell adhesion molecules (CAMs), chemokine signaling, and cytokine-cytokine receptor interaction pathways (Fig. 3F).

### Correlation between LCP1 expression and chemokines or chemokine receptors

**Fig.4:**
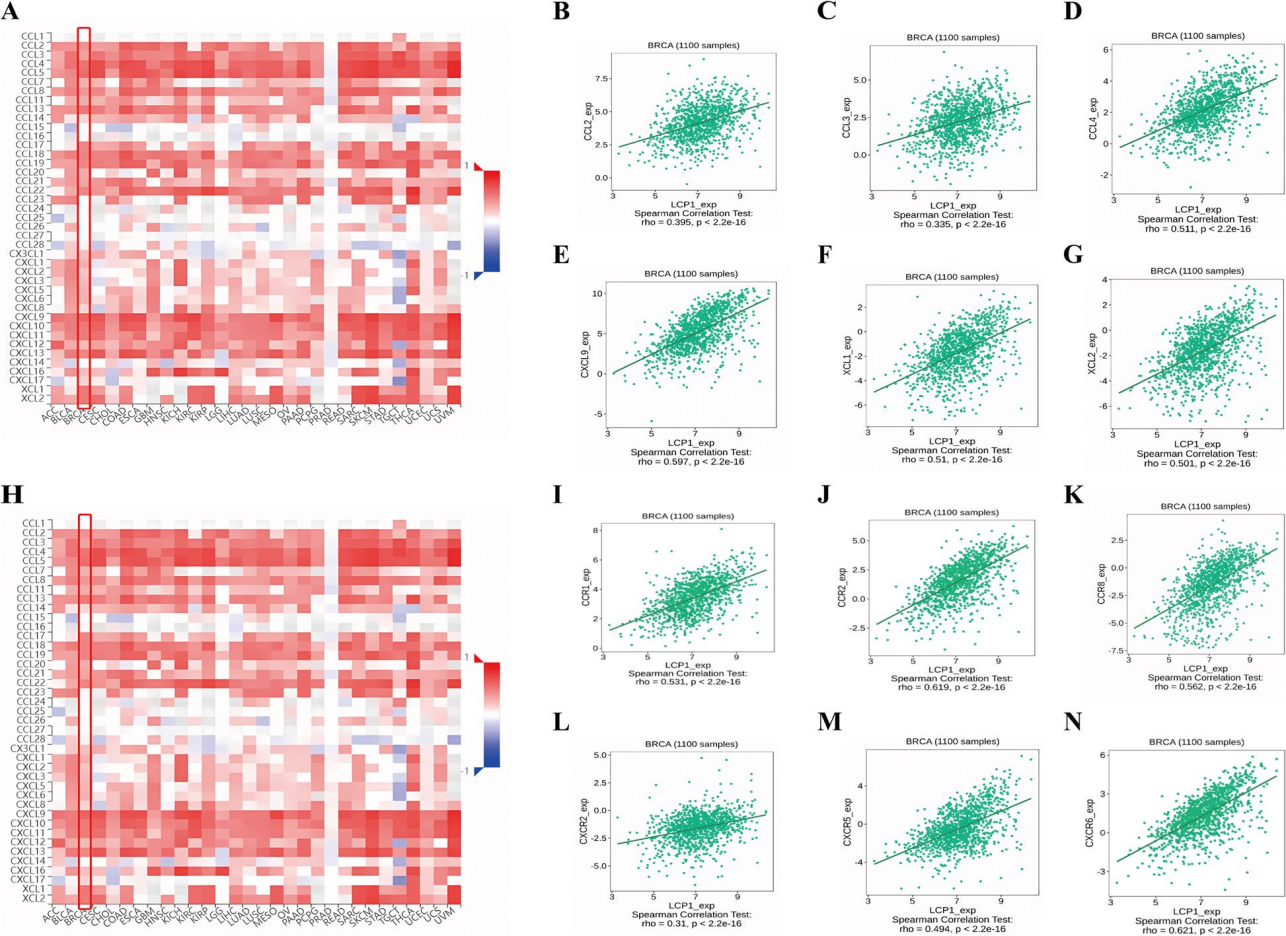
Correlation of LCP1 Expression with Chemokines and Chemokine Receptors in Breast Cancer (BC) Analyzed Using the TISIDB Database. **A** Association between LCP1 expression and various chemokines across 30 tumor types from the TCGA database. **B-G** Specific correlations of LCP1 expression with chemokines CCL2, CCL3, CCL4, CXCL9, XCL1, and XCL2 in the TCGA-BRCA dataset. **H** Relationship between LCP1 expression and diverse chemokine receptors in 30 tumor types from the TCGA database. **I-N** Correlation of LCP1 expression with chemokine receptors CCR1, CCR2, CCR8,CXCR5, CXCR2 and CXCR6

The inflammatory response is intricately modulated by a network of chemokines, which are pivotal in orchestrating immune cell infiltration. Our study delves into the association between LCP1 expression and chemokine dynamics, utilizing the TISIDB database for analysis. We discovered a significant positive correlation between LCP1 expression and various chemokines, namely CCL2(*r*= 0.359, *P*<0.001), CCL3(*r* = 0.335, *P*<0.001), CCL4(*r* = 0.511, *P*<0.001), CXCL9(*r*= 0.597, *P*<0.001), XCL1(*r* = 0.51, *P*<0.001) and XCL2(*r* = 0.501, *P*<0.001) in BC (Fig. 4A-G). Moreover, LCP1 expression also demonstrated a positive correlation with several chemokine receptors, including CCR1(*r* = 0.531, *P*<0.001), CCR2(*r* = 0.619, *P*<0.001), CXCR5(*r* = 0.494, *P*<0.001), CCR8(*r* = 0.562, *P*<0.001),CXCR2(*r* = 0.31, *P*<0.001) and CXCR6(*r* = 0.621, *P*<0.001) (Fig. 4H-N).

### Correlation between LCP1 and immune cells infiltration

**Fig. 5:**
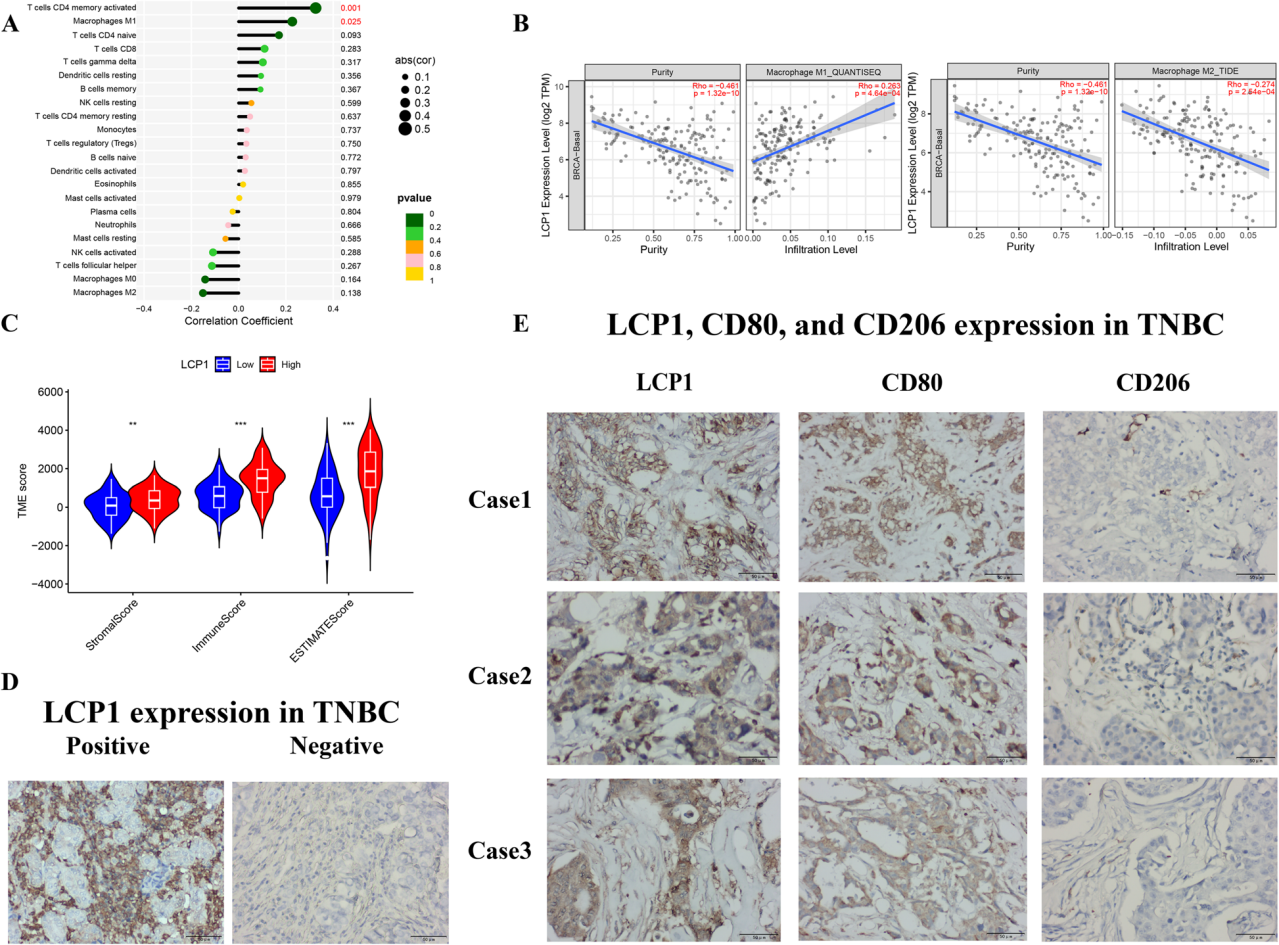
Analysis of the associations of LCP1 with immune cell infiltration. **A** Lollipop diagrams show the correlation between LCP1 expression and immune cell infiltration. **B** Analysis of the correlation between LCP1 expression and M1/M2 macrophages(based on data from TIMER2.0 http://timer.cistrome.org/) **C** The distribution of ImmuneScore, StromalScore and ESTIMATEScore in the low and high LCP1 expression subgroups. **D** The expression of LCP1 in TNBC **E** Immunohistochemical expression of LCP1, CD80 (M1 marker), and CD206 (M2 marker) in three TNBC cases (Note: CD80 serves as a marker for M1 macrophages, while CD206 is indicative of M2 macrophages).*, *P* < 0.05; **, *P* < 0.01; and ***, *P* < 0.001

By applying the CIBERSORT algorithm, it was found that the expression level of LCP1 is positively correlated with the infiltration of M1 macrophages(*P=0.025*) and memory CD4+ T cells(*P=0.001*), and significantly negatively correlated with the infiltration of M2 macrophages(*P=0.138*) in TNBC (Fig. 5A). Analysis from the TIMER database(*n*=191) further confirmed the positive correlation between LCP1 expression and M1 macrophage infiltration(*r*=0.263 *P<0.001*) and its negative correlation with M2 macrophage infiltration(*r*=-0.274 *P<0.001*) (Fig. 5B ,Supplementary Fig. 3). These findings were further validated through IHC, with Fig. 5D displaying negative and positive expression results of LCP1 in TNBC samples. Observing the expression of LCP1, CD80 (M1 marker), and CD206 (M2 marker) in three representative TNBC cases, we found that high expression of LCP1 is usually accompanied by high expression of M1 macrophages and low expression of M2 macrophages (Fig. 5E). Further analysis using the ESTIMATE algorithm indicated that high expression of LCP1 is significantly associated with higher ImmuneScore, StromalScore and ESTIMATEScore in TNBC samples (Fig. 5C), suggesting that high expression of LCP1 may reflect a more active tumor microenvironment.

### Predictive value of LCP1 in immunotherapy

**Fig. 6:**
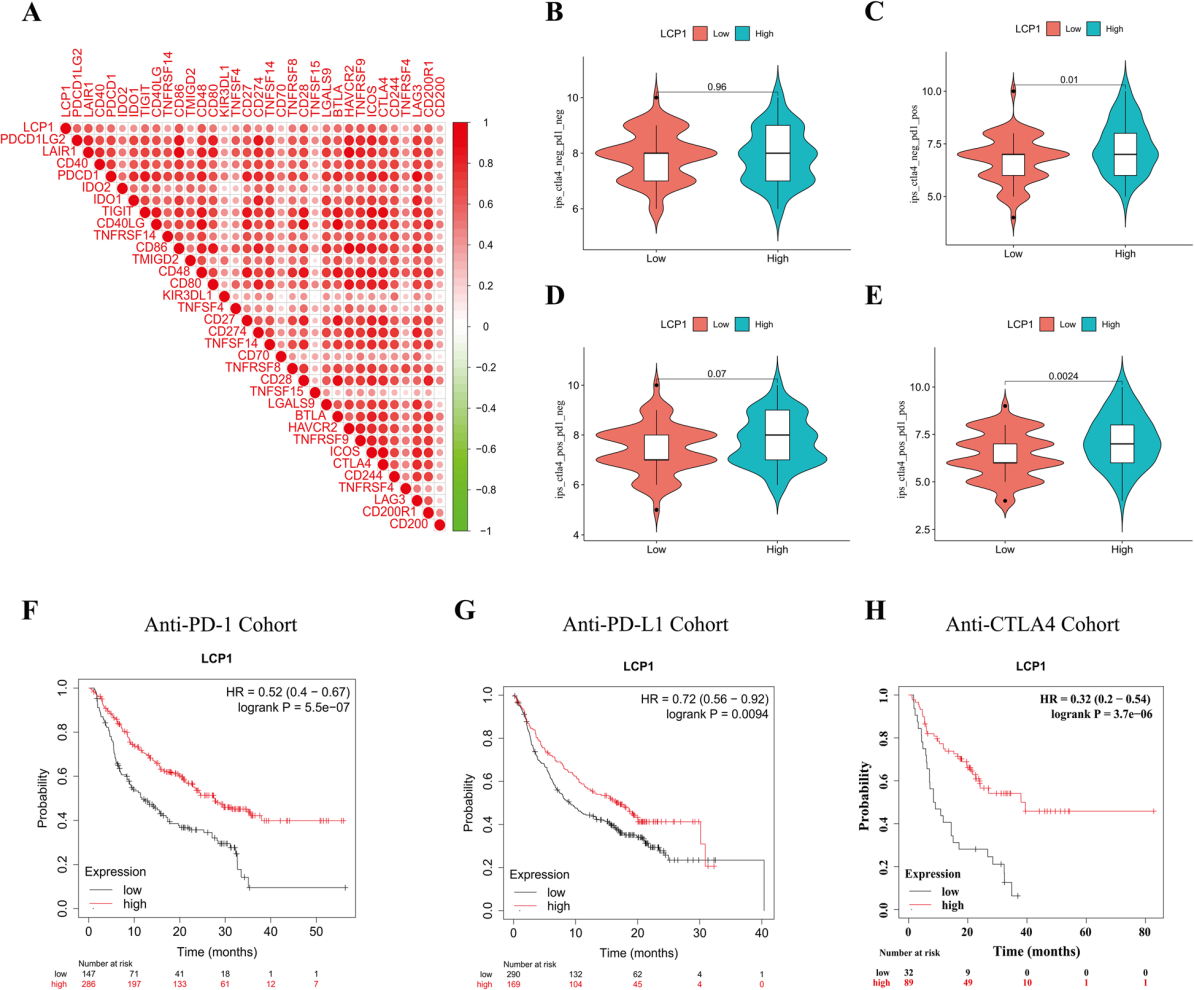
Predictive value of LCP1 in immunotherapy. **A** Analysis of correlations between LCP1 expression and various immune checkpoint genes. **B-E** Comparison of the Immune Phenotype Score (IPS) distributions, differentiated by CTLA-4 or PD-1 status, in high vs. low LCP1 expression groups within the TCGA-TNBC cohort. **F** Kaplan–Meier survival analysis showing the impact of LCP1 expression levels on overall survival in cancer patients treated with anti-PD-1 therapy. **G** Kaplan–Meier survival analysis for overall survival based on LCP1 expression in patients receiving anti-PD-L1 therapy. **H** Kaplan–Meier survival analysis for overall survival depending on LCP1 expression in patients treated with anti-CTLA-4 therapy

**Fig. 7:**
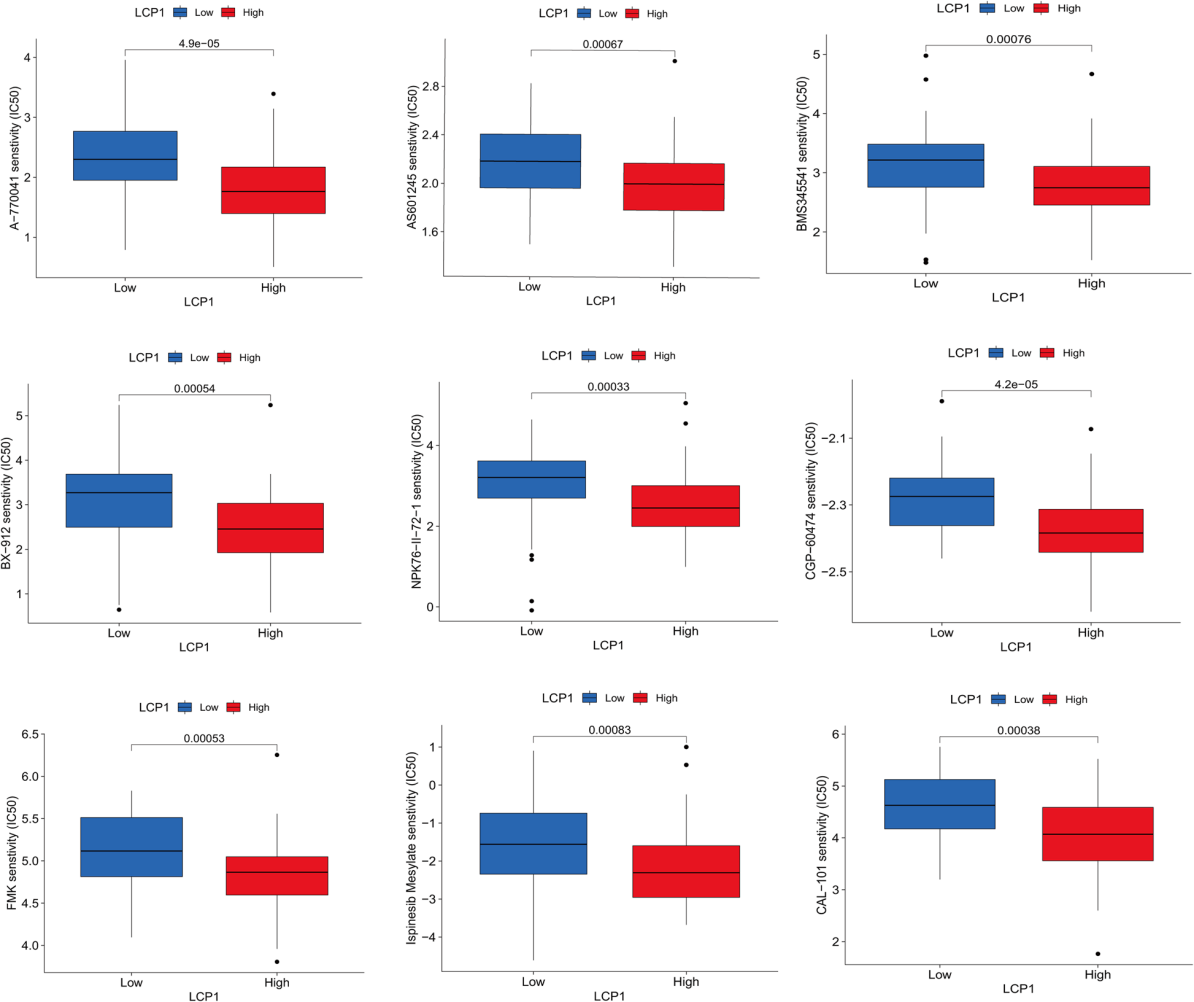
Analysis of drug sensitivity associated with LCP1 expression

Our research delved into the potential involvement of LCP1 in the immune regulation , particularly its impact on immune cell infiltration. Hence, we analyzed the predictive utility of LCP1 for the efficacy of immune therapy, observing significant correlations between LCP1 expression and various immune checkpoint genes (including CD48, TIGIT, TNFRSF4, and ICOS), which show positive correlations with LCP1 expression (Fig. 6A). Moreover, We also applied the Immune Phenotype Score (IPS) to predict tumor responses to immune therapy, especially for treatments targeting CTLA-4 and PD-1. By examining the IPS scores from the TCGA-TNBC dataset, their distribution across different PD-1 or CTLA4 statuses was analyzed. In PD-1 negative cases, the statistical variance related to LCP1 expression was not significant (Fig. 6B, D). However, in PD-1 positive cases, high LCP1 expression was significantly associated with increased IPS scores(*P*=0.01, *P*=0.0024) (Fig. 6C, E). Extending our analysis to a pan-cancer cohort receiving immune therapy, patients with high LCP1 expression generally showed better survival outcomes with anti-PD1(*P<0.001*), anti-PD-L1(*P<0.001*) or anti-CTLA4 (*P<0.001*) immune therapy (Fig. 6F-H). Lastly, through drug sensitivity analysis, chemicals potentially effective for TNBC patients with high LCP1 expression were identified. Utilizing data from the GDSC database, these patients were found to have increased sensitivity to a series of compounds(including A-770041(*P<0.001*),AS601245(*P<0.001*),FMK(*P<0.001*),CGP-60474(*P<0.001*),CAL-101(*P<0.001*),BMS345541(*P<0.001*), NPK76-II-72-1(*P<0.001*) ,BX-912(*P<0.001*), and Ispinesib(*P<0.001*))(Fig. 7).

## Disscussion

LCP1 was initially isolated from fibroblasts of tumor patients and subsequently detected in various cell lines and solid tumor samples [40, 41]. Accumulating research has revealed that the upregulation of LCP1 expression is closely associated with the development of a wide range of human tumors, including colorectal cancer, prostate cancer, breast cancer, and oral cancer [20, 21, 42–44]. Notably, its expression level is directly proportional to the advanced staging and severity of conditions such as colon cancer and breast cancer, thus being regarded as a potential prognostic biomarker [43, 45]. Nonetheless, the specific role of LCP1 in TNBC remains unclear and warrants further exploration. The study focused on assessing the potential of LCP1 as a prognostic indicator for TNBC. Through a detailed analysis of LCP1's expression patterns in normal versus TNBC tissues, its potential role in the prognosis, clinical relevance, and functional enrichment analysis of TNBC was further explored. Results showed that LCP1 is highly expressed in blood, spleen, and lung tissues, while its expression in skeletal muscle is relatively low. Interestingly, its expression in the brain, thyroid, and adipose tissue significantly varies between genders, whereas no significant gender difference was observed in breast tissue. In BC tissue samples, LCP1 expression levels were significantly higher than in normal breast tissue, especially in basal and HER2-positive subtypes. Moreover, compared to normal and luminal subtypes, the protein expression levels of LCP1 were also upregulated in TNBC and HER2-positive breast cancer samples. Recent studies have identified elevated levels of PD-L1 and tumor-infiltrating lymphocytes in TNBC and HER-2 positive breast cancer. TNBC is now recognized as the most immunogenic subtype of breast cancer [46, 47]. Furthermore, LCP1 is highly expressed in both subtypes, indicating its possible role in the tumor's immune processes. These findings underscore the potential key role of LCP1 in the pathogenesis of TNBC, particularly in specific subtypes and immune environment contexts.

Our investigation expands on the link between LCP1 expression levels and patient prognoses in TNBC. The analysis indicates that compared to individuals with high LCP1 expression levels, TNBC patients with low LCP1 expression exhibit lower overall OS, DFS and PFS, highlighting the potential value of LCP1 as a positive prognostic indicator. Similarly, Pillar N et al. have reported that the combination of ABCE1 and LCP1 can significantly inhibit tumor development, reduce metastatic activity, and significantly improve survival rates [48]. In colorectal cancer, the related mechanisms of LCP1 demonstrate a potential positive effect on improving the prognosis of colorectal cancer liver metastasis [49]. However, the expression patterns of LCP1 in other types of cancer differ; in cancers such as cholangiocarcinoma, gastric cancer, and melanoma, high expression of LCP1 is closely related to poor prognosis [23, 50, 51]. The aforementioned studies suggests that LCP1 may play different biological roles in different cancers, emphasizing the necessity of a detailed assessment of its application as a biomarker for specific cancer types. Notably, the expression levels of LCP1 are not significantly related to the patient's age, cancer stage, tumor size, or lymph node involvement. The results are consistent with the analysis of the TCGA database. This may be due to the small sample size. Further studies with a larger sample size are needed to validate the relationship between LCP1 and the clinical characteristics of patients.

We delved deeply into the biological functions of LCP1 in TNBC. Functional enrichment analysis revealed that LCP1 is closely associated with numerous immune-related genes and pathways, particularly playing a significant role in immune signaling, hematopoietic cell lineage, cell adhesion molecules pathways, and immune system functions. LCP1 promotes cell migration and immune response by facilitating actin polymerization, cytoskeletal reorganization, and phagocytosis [52]. Moreover, LCP1 is closely associated with ATP synthesis, oxidative phosphorylation, and mitochondrial membrane proteins, playing a pivotal role in cellular energy production and migration pathways [53]. A study revealed significant effects of LCP1 gene knockdown on immune cell behavior. Specifically, the knockdown of LCP1 triggered changes in the phosphorylation patterns of key signaling kinases and transcription factors within monocytic-derived macrophages (MoDMs), which were closely related to genes involved in fatty acid metabolism and glycolysis. Moreover, the study also found a correlation between LCP1 expression levels and the migration capabilities of immune cells, further highlighting the crucial role of LCP1 in immune regulation [53]. Although there are currently no studies reporting the mechanisms by which LCP1 regulates the immune system, one study indicates that its family member, LCP2, participates in the activation of TCR signaling and indirectly influences TCR signaling through CD28 and the B7 family, thereby modulating anti-tumor immunity response [54]. Concurrently, we observed that the enrichment of migration-related genes is correlated with LCP1 expression levels, which is consistent with the results of the aforementioned study. Studies indicated that LCP1 is crucial for the migration potential of lymphocytes and certain tumor cells, further proving LCP1's key regulatory role on cell morphology and behavior in adaptive and innate immune systems (including macrophages, B cells, T cells, and NK cells) [16, 17, 55–57]. Thus, these results displayed LCP1 multifunctionality in TNBC, especially its critical role in regulating immune responses.

The tumor microenvironment (TME) is composed of tumor cells, pericytes, macrophages, endothelial cells, fibroblasts, and non-cellular components such as the extracellular matrix (ECM) and soluble signals [58]. Tumor-infiltrating lymphocytes (TILs) are a crucial component of the TME, playing a key role in recognizing and eliminating tumor cells. Other components of the TME, such as cytokines and chemokines, can influence the activity and function of TILs [59]. By exploring the association between LCP1 expression and chemokines and their receptors, we found a significant positive correlation between LCP1 and various chemokines and receptors. Research by Jason et al. highlighted LCP1's importance in leukemia cells' reactive migration to CXCL12, emphasizing its potential role in regulating immune cell infiltration and inflammatory responses [60]. Another research suggests that LCP1 regulates lymphocyte polarity and migration by enabling cells to establish an axis of asymmetry in response to chemokine signaling [61]. Furthermore, Our results by immunohistochemistry uncover a noteworthy association between elevated LCP1 expression and the increased prevalence of M1 macrophages, coupled with a reduced expression of M2 macrophages. Within the TME, heightened levels of activated memory CD4+ T cells and M1 macrophages emerge as biomarkers indicative of a positive prognosis [62]. A study confirmed that LCP1 might influence cell shape by affecting cytoskeletal changes, leading to the polarization of macrophages from the M1 to the M2 phenotype [63]. Recent evidence showed that TILs have been observed in HER2-positive breast cancer and TNBC patients, and TNBC cases marked by significant lymphocyte infiltration demonstrated enhanced long-term survival rates and a superior response to chemotherapy [64]. Thus, breast cancer is no longer considered an immunologically quiescent tumor type. Hence, we suggest that the prognostic relevance of LCP1 primarily lies in its immunological impact within TNBC. However, the mechanisms by which LCP1 influences macrophage polarization still require further experimental exploration.

Previous studies have shown that the abundance of TILs in the tumor microenvironment is associated with a better response to immune checkpoint inhibitors (ICI) therapy [65, 66]. Facing the moderate efficacy of PD-1/PD-L1 immune checkpoint inhibitors (ICIs) in TNBC, our findings suggest a positive correlation between LCP1 and high expression of checkpoint genes, indicating that patients with high LCP1 expression may be more suitable for ICIs treatment. Based on previous research, IPS is considered a useful biomarker for predicting patients' response to immunotherapy. IPS can quantify the determinants of tumor immunogenicity and has predictive value in cancer patients treated with CTLA-4 and PD-1 blockers [67]. Currently, Atezolizumab and Pembrolizumab have been approved for use in combination with chemotherapy in patients with PD-L1-expressing unresectable locally advanced or metastatic TNBC [68, 69].In this study, we further validated the predictive value of LCP1 through the ESTIMATE algorithm, IPS predictors, and public immune therapy cohorts, suggesting its high expression may serve as an important marker for assessing the efficacy of immunotherapy, especially in patients with positive PD-1 expression. Finally, through drug sensitivity analysis, we identified compounds potentially effective for patients with high LCP1 expression in TNBC. Compounds such as A−770041, AS601245, FMK, CGP−60474, CAL−101, BMS345541, BX−912, NPK76−II−72−1, and Ispinesib are considered potential drugs targeting LCP1. This suggests that patients with high LCP1 expression may benefit from chemotherapy, which can stimulate an immune response by increasing the antigenicity of cancer cells or enhancing their adjuvant properties, defined as a potential mechanism of immunogenic cell death. Currently, no studies have directly reported any of these drugs in relation to LCP1. These drugs may contribute to the development of BC candidate drugs and provide a scientific basis for personalized treatment based on LCP1 expression.

Our study has identified the prognostic significance of LCP1 in patients with TNBC, from the dual perspectives of bioinformatics and immunohistochemical validation, to determine its viability as a biomarker. However, the study faces several noteworthy limitations. First and foremost, our survival data rely on public databases, underlining the need for clinical samples with comprehensive long-term survival data and prospective cohorts of TNBC patients receiving ICIs treatment for the affirmation of LCP1 prognostic merit in TNBC. Additionally, the mechanisms and functional roles of LCP1 in remodeling the TNBC immune microenvironment both in vivo and in vitro warrant further detailed exploration. Lastly, the analytical approaches employed herein are capable of indicating potential correlations, necessitating further experimental corroboration in future endeavors.

## Conclusion

In conclusion, our research is based on the analysis of public databases and immunohistochemical analyses. High expression of LCP1 correlates with enhanced immune cell infiltration and prolonged survival in TNBC patients. The results of the present study highlight the promising potential of LCP1 as both a diagnostic and prognostic biomarker, underscoring its significance in the therapeutic landscape of TNBC.

### Supplementary Information

Additional file 1: Supplementary Fig.1 The LCP1 expression of various human tissues in males and females. *, *P* < 0.05; **, *P *< 0.01; and ***, *P *< 0.001. Supplementary Fig. 2 Genetic Alterations of LCP1 in the TCGA-BRCA Cohort A An OncoPrint visual summary of alterations in LCP1 within the TCGA-BRCA cohort. Five types of genetic alterations were identified: missense mutation (of unknown significance), amplification, deep deletion, mRNA high expression, and mRNA low expression. B A comprehensive overview of the frequency of LCP1 alterations based on TCGA-BRCA mutation data. C. Analysis of gene mutation co-occurrence between the altered and unaltered groups of the LCP1 gene. Supplementary Fig. 3 The correlation between LCP1 and the infiltration of M1 and M2 macrophages using different algorithms. Supplementary Table 1. The DEGs analysis of LCP1 in TNBC.

## Data Availability

The datasets generated and/or analysed during the current study are not publicly available due to privacy restrictions but are available from the corresponding author on reasonable request.
